# 1409. Infectious Complications in People Who Inject Drugs (PWID): A Retrospective Review and Recommendations for Improved Care

**DOI:** 10.1093/ofid/ofac492.1238

**Published:** 2022-12-15

**Authors:** Elena K Martin, Caroline Wang, Audun J Lier, Nancy Azab, Bettina F Fries

**Affiliations:** Renaissance School of Medicine at Stony Brook University, Stony Brook, New York; Renaissance School of Medicine at Stony Brook University, Stony Brook, New York; Northport VAMC, Northport, New York; Renaissance School of Medicine at Stony Brook University, Stony Brook, New York; Renaissance School of Medicine at Stony Brook University, Stony Brook, New York

## Abstract

**Background:**

Suffolk County, NY is home to 1.5. million people and has a higher drug related deaths per capita rate compared to NY state average (19.9 vs 14.9/100,000). Injection drug use (IDU) is associated with complicated infections and inadequate treatment of substance use disorder (SUD) leads to relapse of infection and treatment failure. Unfortunately, access to SUD treatment services is not optimized in Suffolk County. We aimed to characterize the incidence and outcome of admissions for PWID and identify modifiable risk factors which can contribute to suboptimal care for PWID.

**Methods:**

A retrospective review of SBUH electronic medical record was conducted from Jan 1, 2015 to June 1, 2021. Length of stay, discharge disposition, 6-month hospital readmission, insurance, employment and housing status, SUD history plus infection type and antimicrobial receipt were assessed.

**Results:**

Of 425 patients (pts) admitted between Jan 1, 2015 to June 1, 2021, the median age was 34 years, 93% were white, and 59% male. Most pts. (89%) reported stable housing and had government insurance. The average length of stay was 13.6 days with 1/3 of pts. requiring ICU admission. Notably, 34% of pts left prematurely against medical advice and 34% were readmitted within 6 months. Formal ID (63%) and Psychiatry (62.5%) consults were not requested on all pts. Only 18% and 25% of pts. were treated with methadone or suboxone. 60% of screened pts. had HCV but 50% were not screened for co-infections. Complicated skin and soft tissue represented 70%, sepsis/bacteremia 11%, osteomyelitis/arthritis 10% and endocarditis 12% of the cases. 14.8% of pts. with endocarditis died, 48% had recurrent endocarditis, 52% needed surgery including 12% of repeat valve replacements. *Staphylococcus* species caused 42% of infections. About 2/3 of pts required continued antibiotic therapy at time of discharge, leading to delayed discharge for IV catheter placement.

**Conclusion:**

Managing infectious complications in PWID is a multi-faceted endeavor extending beyond inpatient care. This study highlights the need for standardized treatment plans involving specialized case managers to reduce LOS and AMA discharges, improve screening for infections and integrate SUD treatment for PWID.

**Disclosures:**

**All Authors**: No reported disclosures.

